# Individual and community-level determinants of cervical cancer screening in Zimbabwe: a multi-level analyses of a nationwide survey

**DOI:** 10.1186/s12905-022-01881-0

**Published:** 2022-07-25

**Authors:** Alone Isabirye, Bob Charlestine Elwange, Kavita Singh, Manuela De Allegri

**Affiliations:** 1 Department of Sociology, Anthropology, and Population Studies (Demography), Faculty of Social Sciences, Kyambogo University, Kampala, Uganda; 2 Heidelberg Institute of Global Health, University Hospital and Faculty of Medicine, University of Heidelberg, Im Neuenheimer Feld 130.3, 69120 Heidelberg, Germany; 3 Public Health Foundation of India, New Delhi, India

**Keywords:** Screening, Cervical cancer, Zimbabwe

## Abstract

**Background:**

Despite the benefits of cervical cancer (CC) screening to reduce the disease burden, uptake remains limited in developing countries. This study aims to assess the individual and community-level determinants of cervical cancer screening among women of reproductive age in Zimbabwe.

**Methods:**

We analyzed data collected from 400 communities from the 2015 Zimbabwe Demographic and Health Survey with a sample size of 9955 women aged 15–49 years. The descriptive statistics and multi-level regression models adjusted for potential covariates were performed to examine the association between individual, household and community-level factors and the uptake of cervical cancer screening in women.

**Results:**

The mean (SD) age of women in Zimbabwe using cervical cancer screening was 27.9 (9.9) years. A relatively small proportion of women, i.e., only 13.4% had ever screened for cervical cancer, with higher screening rates observed in the following sub-groups: middle aged women 31–49 years (odds ratio (OR) = 2.01; 95% confidence intervals (CI) 1.72–2.34), and currently working (OR = 1.35; 95% CI 1.17–1.55), those with health insurance (OR = 1.95; 95% CI 1.63–2.34), used modern contraceptives (OR = 1.51; 95% CI 1.22–1.86), exposed to multiple media (OR = 1.27; 95% CI 1.03–1.58), those living in communities that had a high predominance of women with favorable attitude towards Intimate Partner Violence (IPV) against women (OR = 1.21; 95% CI 1.04–1.41) and a non-poor wealth index (OR = 1.54; 95% CI 1.14–2.05).

**Conclusions:**

Our data shows a significantly low prevalence of cervical cancer screening among reproductive age women in Zimbabwe. To increase the uptake of cervical cancer screening, there is an urgent need both to implement behavioral interventions targeted at women from low socio-economic groups and to advocate for universal health coverage that includes financial risk protection to help all women realize their right to health.

## Background

Cervical cancer is a major public health concern with a disproportionately higher disease burden (84% of all incident cases and 87% deaths related to cervical cancer) in low-and middle-income countries (LMICs) [1]. In high-income countries, widespread cytology-based screening has resulted in a decrease in both the incidences of cervical cancer and the mortality rate [2], whereas in LMICS, due to poor screening access and a low uptake, the number of incidences and the mortality rate continue to rise [3]. For instance, the number of cervical cancer cases in Zimbabwe increased from 542 in 2008 to 1,368 in 2015 [4]. The sub-Saharan Africa region has an age-standardized incidence rate of 43 per 100,000 and a mortality rate of 20 compared with the global estimate of 14.0 and 6.8 respectively [5]. Among countries in Southern Africa, Zimbabwe carries the highest burden, with an age-standardized incidence rate of 62 per 100,000 women, i.e., an incidence rate five times higher than the global average. Furthermore, the age-standardized mortality rate of 46 per 100,000 women in Zimbabwe is nearly seven times the global average [6]. The 2018 estimates indicated that about 3,186 women were newly diagnosed with and 2,151 died due to cervical cancer in Zimbabwe [7].

To reduce the burden of cervical cancer in LMICs, both HPV vaccination and regular cervical cancer screening efforts are recommended. However, substantial “know-do” gaps exist due to health system-, provider- and patient-level factors [8]. The World Health Organization (WHO) recommends that women initiate cervical cancer screening at 30 years of age [9] because of the elevated risk of HPV-positivity in the age-group. By targeting this age group, screening would be beneficial to women, even if done once [9]. The cervical cancer screening methods involve simple and convenient visual tests [with Lugol's iodine (VILI) or acetic acid (VIA)], the Papanicolaou test (Pap smear), and HPV-DNA testing [10]. Despite the differences in the screening approaches, they are all proven to be effective for cervical cancer screening [10, 11]. Of the four screening methods, the HPV-DNA test is highly sensitive and convenient (self-collection of samples). However, the HPV-DNA test cannot be used for detecting pre-cancer cells; it has to be followed by one of the visual tests, especially when the HPV-DNA result is positive [12]. This is why the WHO recommend the use of VIA/VILI in resource limited settings because the HPV-DNA test is not only affordable, but women can also be screened and treated in a single visit [10, 11]. Furthermore, the WHO recommend HPV-DNA testing followed by the visual tests in settings where staged screening can be sustained economically [12].

However, cervical cancer screening remains available only in selected public health facilities in the African region, which presents obvious access challenges [13] to a primarily rural population. Although private health facilities in Zimbabwe complement public health facilities in providing cervical cancer screening services, they are not easily accessible due to high treatment costs [13], particularly for the poor and rural population [14]. In 2012, Zimbabwe adopted Visual Inspection with Acetic Acid and Cervicography (VIAC) in public health facilities [15]. At these facilities, women who test/screen VIAC positive are treated immediately. Methods including loop electrosurgical excision procedure (LEEP), cauterization, and cryotherapy are used under the see and treat model. Women with potential cancer lesions are referred to the next level of care for biopsies [15]. The 2018 Ministry of Health goal was to screen 25% of the women aged 25–59 years [13]. In general, Zimbabwean women have shown high levels of awareness [16, 17] and expressed positive attitudes towards cervical cancer screening [18, 19] despite challenges in healthcare access [15]. However, suboptimal care exists as indicated by prior studies that show a very low uptake of cervical cancer screening ranging from 5 to 34% in Zimbabwe [17–21]. Another study reported a decline in the uptake of VIAC between 2014 and 2016 [22].

To inform clinical practice and policies for the prevention of cervical cancer, there is an urgent need to generate empirical evidence on the population level factors or determinants of cervical cancer screening [23–26]. However, limited data exists on the individual and community level determinants of the utilization of cervical cancer screening opportunities in Zimbabwe. Previous research has indicated that negative religious beliefs [17, 18], inadequate knowledge regarding cervical cancer screening [19], and the unavailability of screening services at women’s usual points of health care [17] play a critical role and influence the uptake of cervical cancer screening. However, existing studies thus far are limited by a small sample size, based on data derived from facility-based surveys, lacked national scope/relevance, and did not account for community level factors. This study aims to assess the association between individual, household, and community level factors and the uptake of cervical cancer screening among women of reproductive age in Zimbabwe using multi-level regression analysis. The findings of this study will guide the design and effective implementation of strategies to reduce cervical cancer burden in Zimbabwe.

## Methods

### Study design and data sources

The study is a cross-sectional analysis of the secondary data from the 2015 Zimbabwe Demographic and Health Survey (ZDHS). The ZDHS is a nationally representative cross-sectional survey that applied a stratified two-stage cluster sampling design [27], drawing from the 2012 population and housing census [28]. In the first stage, enumeration areas (EA)/villages/clusters were randomly selected, followed by households in the second stage. Female household members age 15–49 years who were either permanent residents or visitors a night to the survey were selected [27]. A detailed description of the sampling approach is published in the ZDHS report [27]. Data of 9,955 women drawn from ten provinces were included in this study. The ten provinces included Manicaland (1,019 women), Mashonaland central (993 women), Mashonaland East (910 women), Mashonaland West (1,054 women), Matabeleland North (849 women), Matabeleland South (829 women), Midlands (1,062 women), Masvingo (1,046 women), Harare (1,235 women), and Bulwayo (658 women) [27]. The sub-samples from the provinces were based on the proportionality of the number of women in each province.

### Outcome measure

The primary outcome measure “have ever screened for cervical cancer” was measured using the self-reported question: “Have you ever been screened for cervical cancer?” and the response was coded as a binary variable: 1 if yes and 0 if no. This question was asked to all women aged 15–49 years in the ZDHS conducted in 2015 in Zimbabwe.

### Exposure/explanatory variables

We examined the possible association between individual and community level factors and “ever screened for cervical cancer” among women of reproductive age living in Zimbabwe. The selection of both individual-level and community-level variables were based on the previously reported studies and the available relevant variables in the ZDHS dataset [20, 29–32].

*Individual-level variables* considered in the analysis were women’s age, religion, employment status, health insurance coverage, region, contraceptive use, total children ever born, type of marriage, household size, and place of delivery. Furthermore, we classified the exposure variables based on previous reports [20, 31, 32]. The participant’s age was categorized into two groups: < 30 and 31–49 years. Religion was classified into Christians and non-Christians, and highest education attainment was classified as less than primary school, secondary school, and high school or Graduates. Employment status, covered by health insurance, and “ever used modern contraceptives” were coded as binary variables with responses 1 = Yes and 0 = No. The total children born were classified into ≤ 3 and ≥ 4 children, age at first sex was classified into < 17, 18–22 and 23–37 years; type of marriage was classified as monogamy, polygamy or not in union; source of media exposure was grouped as single, multiple or none. The participant’s household size was grouped into 1–4 persons, 5–8 and > 9 family members. The place of delivery of new-born was classified as health facility, home or never given birth. The 2015 ZDHS dataset captured ten regions, which were classified into six categories (Manicaland, Mashonaland, Matabeleland, Midlands, Masvingo, Harare, and Bulwayo), regions with homogenous characteristics were merged and assigned one category. Wealth index was a composite score pre-measured by household assets such as televisions, bicycles, materials used for house construction, water access types, sanitation facilities, and other characteristics related to wealth index. Factor scores of household assets were generated through a principal component analysis and were then standardized and categorized into quintiles (poorest, poorer, middle, richer, and richest) [27].

The *community level determinants* were conceptualized as a set of variables capturing community disadvantages, i.e., the factors that make it difficult for people living in certain areas to achieve positive life outcomes. The socio-economic indices for areas (SEIFA) approach was used to identify community factors leading to disadvantages/advantages [33]. With the exception of type of residence, the 2015 ZDHS did not collect extensive data on community measures. Therefore, women’s individual level responses were aggregated to construct factors causing community disadvantages. The community variables were defined based on the aggregate responses of women in relation to the 400 communities considered by the 2015 ZDHS. The specific community level measures included women’s decision-making autonomy, women’s attitudes towards domestic violence (wife-beating), type of residence, perceived distance/access to a health facility, and community-level socioeconomic status. The aforementioned variables have been derived and used as measures of community disadvantages by studies elsewhere [34, 35]. The decision-making power or autonomy in the household was measured using the responses to the following three questions: who decides matters pertaining to (a) the woman’s health (personal decision-making authority), (b) visits to friends or family (mobility decision-making authority), and (c) food to be cooked each day. First, we generated an individual-level indicator by differentiating women who made all three decisions, either alone or jointly with their spouses, as having “high decision-making autonomy”, from women who did not as having “low decision-making autonomy”. Second, we aggregated the scores of individuals at the community-level to derive the proportion of women with high decision-making autonomy for every community/cluster. Women’s justification of Intimate Partner Violence (IPV) against themselves is a measure of disempowerment, and was associated to reproductive health outcomes in Uganda [36]. Women’s justification of IPV towards themselves was assessed by asking women if they believed that a man had a right to beat his wife for five hypothetical scenarios: (1) she goes out without telling him, (2) she neglects the children, (3) she argues with him, (4) she refuses to have sexual intercourse with him, and (5) she does not cook food properly. Similarly, as described above, we first classified individual responses, differentiating women as having a favorable attitude towards IPV when responding positively to at least one of the five scenarios and as having a negative attitude towards IPV otherwise. Then, we aggregated values at the community-level to derive the proportion of women with a favorable attitude towards IPV. This approach of measuring women’s justification of IPV against themselves was used by previous studies in Kenya [34] and Uganda [36]. The 2015 ZDHS posed a question “Do you perceive distance to a health facility to be a big challenge” with no/yes responses. We used this variable as a proxy-measure for community disadvantage in terms of access to health facilities. The percentage of women who responded that distance was a big challenge in accessing health facilities was established and the total number per community obtained. For the 400 communities, the minimum score was 0, and the maximum was 27, with a mean score of 7. Clusters that had scores above the mean were categorized as communities with a higher proportion of women who reported distance to the health facility as a big challenge (coded 0) and vice-versa. Furthermore, the wealth indices for women were aggregated at their respective villages to obtain aggregate community socio-economic disadvantages. This was done by classifying women with middle, richer and richest wealth indices as having a non-poor wealth index and proportions for each community were established. The community-level socioeconomic measure was obtained by categorizing clusters into those with high and low proportions of women with a non-poor wealth index. This approach of measuring socioeconomic disadvantages has been used by studies in Kenya [34] and Korea [37]. The composition of group-constructs from an individual-level survey dataset is beneficial, especially where multi-level analytical models [38] are deployed to provide evidence regarding the contribution of community-level factors [33].

### Statistical analyses

We used descriptive analysis to report women’s demographic and socioeconomic characteristics. Next, we conducted cross-tabulation and applied Pearson’s chi-squared (${x}^{2}$) tests to investigate associations of individual and community level characteristics with an uptake of cervical cancer screening. Furthermore, the associations of individual- and community-level determinants with an uptake of cervical cancer screening were analyzed in a stepwise manner. The nesting of individuals within communities in which women lived generated three models for analysis. We started by fitting the variance component model or empty model (null model). The empty model excluded the fixed effects. To assess the model fit, we estimated the likelihood ratio test and Akaike Information Criterion (AIC) of the models, with a lower AIC value denoting a better model fit [39]. The odds of cervical cancer screening adjusting for each of the individual-level and community-level determinants in model 3 were presented along with p-values and 95% confidence intervals [40]. We performed Variance Inflation Factor (VIF) and Tolerance tests to check for multicollinearity among the covariates in the models. We did not observe any significant multicollinearity issues in the regression models, since all variance inflation factor values were less than ten and tolerance values were greater than 0.1. The two-tailed Wald test was used to determine the statistical significance of the covariates at a significance level of alpha equal to 5% [41]. All statistical analysis were performed using Stata SE 15 software.

### Ethical considerations

All data used in the study were secondary and obtained in a fully anonymized format from the 2015 ZDHS. Therefore, no participant/institutional ethical approval was required for this study. Data collection was conducted in accordance with the Declaration of Helsinki for conducting research involving humans. During data collection, written informed consent was obtained from both the adult participants and the parent(s)/guardian(s) of all under-16 s prior to the interviews [27]. Procedures and questionnaires for standard DHS surveys have been reviewed and approved by the Institutional Review Board (IRB) of the Inner City Fund (ICF) International. Additionally, we obtained approval to use the data from the DHS repository (http://dhsprogram.com/data/available-datasets.cfm).

## Results

### Descriptive characteristics

The study results revealed that over half of the respondents were $\le$ 30 years (59.4%), in monogamous marital relationships (54.6%), and had attained secondary education (66.7%). Nearly half (49.5%) were not working, and living in households with 5–8 members (45.6%). Most of the women were not covered by health insurance (87. 8%), and had $\le$ 3 children (78.7%). About 1 in 3 were residing in the region of Mashonaland (29.7%), and had never used any form of modern contraception (31.5%). Close to one-third were age $\le$ 17 years when first having sexual intercourse (36. 6%) and nearly all (94.3%) women were Christians. Close to a quarter (24. 2%) did not have access to any form of media and approximately 4 in 10 (41.2%) gave birth from a health facility. Considering community-level variables, over half were residing in communities with high proportions of women; supportive to IPV against women (61. 7%), with non-poor wealth index (51.6%) and urban dwelling (54.6%). Over half of the women were residing in communities with low proportions of women with high decision-making autonomy (56. 2%), and with challenges in accessing health care due to distance (59.5%).The study findings also indicate that few women (13.4%) had ever screened for cervical cancer. Among the poorest, prevalence was slightly lower at 5.1%.

Bivariate analysis revealed a significant relationship between cervical cancer screening and all individual-level factors. The proportion of women who had ever screened was relatively higher among women who were age 31–49 years (p < 0.001), Christians (p < 0.026), had attained post-secondary education (p < 0.001), working (p < 0.001), covered by health insurance (p < 0.001), from Mashonaland region (p < 0.001), using any modern method of contraception (p < 0.001), having $\le$ 4 children (p < 0.001), age 23–37 years at first sex (p < 0.001), the richest (p < 0.001), exposed to multiple sources of media (p < 0.001), in monogamous marital relationships (p < 0.001), living in household with 1–4 members (p < 0.001), and had delivered from health facilities (p < 0.001). The percentage of women who had ever screened for cervical cancer was also relatively higher among women who were living in communities with a high proportion of women with a favorable attitude towards IPV against women (p < 0.001), a non-poor wealth index (p < 0.001), and urban dwellers (p < 0.001). The percentage of women who had ever screened was relatively higher among women who were living in communities with a low proportion of women who reported distance to health facility as a big challenge (p < 0.001) (Table 1).

**Table 1:** Distribution of women by individual and community characteristics by cervical cancer screening status (N = 9955)

Characteristics	Never screened N (%)	Ever screened N (%)	Overall N (%)	p-values
Total	**8617 (86.6)**	**1338 (13.4)**	9955	
Age group				**0.001**
≤30	5457 (92.3)	454 (7.6)	5911 (59.4)	
31–49	3160 (78.1)	884 (21.9)	4044 (40.6)	
Religion				**0.026**
Christians	8106 (86.4)	1279 (13.6)	9385 (94.3)	
Non-Christians	511 (89.7)	59 (10.4)	570 (5.7)	
Education level				**0.001**
≤Primary	2302 (92.4)	189 (7.6)	2491 (25.0)	
Secondary	5757 (86.7)	880 (13.3)	6637 (66.7)	
Post-secondary	558 (67.5)	268 (32.5)	827 (8.3)	
Currently working				**0.001**
No	4543 (92.2)	385 (7.8)	4928 (49.5)	
Yes	4074 (81.0)	953 (19.0)	5027 (50.5)	
Covered by health insurance				**0.001**
No	7800 (89.3)	939 (10.7)	8739 (87.8)	
Yes	817 (67.2)	399 (32.2)	1216 (12.2)	
Region				**0.001**
Manicaland_	927 (91.0)	92 (9.0)	1019 (10.2)	
Mashonaland	2587 (87.5)	370 (12.5)	2957 (29.7)	
Matabeleland	1530 (91.2)	148 (8.8)	1678 (16.9)	
Midlands	965 (90.9)	97 (9.1)	1062 (10.7)	
Masvingo	909 (86.9)	137 (13.1)	1046 (10.7)	
Harare	948 (76.8)	287 (23.2)	1235 (12.4)	
Bulwayo	751 (78.4)	207 (21.6)	958 (9.6)	
Ever used modern contraceptives				**0.001**
No	2988 (95.4)	143 (4.6)	3131 (31.5)	
Yes	5629 (82.5)	1195 (17.5)	6824 (68.6)	
Total children ever born				**0.001**
≤3	6835 (87.2)	1000 (12.8)	7835 (78.7)	
≥4	1782 (84.1)	338 (15.9)	2120 (21.3)	
Age at first sex				**0.001**
Never had sex	1819 (99.7)	6 (0.3)	1825 (18.3)	
≤17	3181 (87.1)	461 (12.7)	3642 (36.6)	
18–22	3121 (82.1)	681 (18.0)	3802 (38.2)	
23–37	496 (72.3)	190 (27.7)	686 (6.9)	
Wealth index				**0.001**
Poorest	1423 (94.4)	76 (5.1)	1499 (15.1)	
Poor	1370 (94.4)	82 (5.7)	1452 (14.6)	
Middle	1422 (91.8)	127 (8.2)	1549 (15.6)	
Rich	2144 (83.9)	414 (16.2)	2558 (25.7)	
Richest	2254 (77.9)	639 (22.1)	2897 (29.1)	
Amount of media exposure				**0.001**
None	2240 (92.8)	173 (7.2)	2413 (24.2)	
One	2874 (88.9)	358 (11.1)	3232 (32.5)	
Multiple	3503 (81.3)	807 (18.7)	4310 (43.3)	
Type of marriage				**0.001**
Monogamy	4471 (82.3)	964 (17.7)	5435 (54.6)	
Polygamy	516 (89.0)	64 (11.0)	580 (5.8)	
Not in union	3630 (92.1)	310 (7.9)	3940 (39.6)	
Household size				**0.001**
1–4	3791 (85.3)	654 (14.7)	4445 (44.7)	
5–8	3946 (86.9)	593 (13.1)	4539 (45.6)	
9+	880 (90.6)	91 (9.4)	971 (9.8)	
Place of delivery				**0.001**
Health facility	3449 (84.1)	650 (15.9)	4099 (41.2)	
Home	698 (95.1)	35 (4.9)	734 (7.4)	
Never given birth	4470 (87.3)	652 (12.7)	5122 (51.5)	
Community level factors				
Women with favorable attitude towards IPV against women				**0.001**
Low	3488 (91.6)	322 (8.5)	3810 (38.3)	
High	5129 (83.5)	1016 (16.5)	6145 (61.7)	
Women with high decision making autonomy				**0.001**
Low	4925 (88.1)	668 (11.9)	5593 (56.2)	
High	3692 (84.6)	670 (15.4)	4362 (43.8)	
Household wealth index				**0.001**
Low	4477 (93.0)	339 (7.0)	4816 (48.4)	
High	4140 (80.6)	999 (19.4)	5139 (51.6)	
Difficulty in access to healthcare				**0.001**
Low	4876 (82.3)	1050 (17.8)	5926 (59.5)	
High	3741 (92.9)	288 (7.2)	4029 (40.5)	
Type of residence				**0.001**
Rural	5009 (92.2)	425 (7.8)	5434 (54.6)	
Urban	3608 (79.8)	913 (20.2)	4521 (45.4)	

Bold results indicate statistically significant p-values

Table 2 depicts the results of the multi-level regression analyses. Results of Model I indicated a high (0.56) and statistically significant (p < 0.001) variance partition coefficient (VPC) or intra-cluster correlation (ICC) indicating, a 56.0% variation in cervical cancer screening as a result of women living in their respective communities and the appropriateness of deploying multi-level rather than individual-level analyses. The appropriateness of deploying multi-level regression analyses is supported by the statistically significant ICC (p < 0.001) that depicts the dependence in the data structure. The results of the Variance Component Model also provided estimations of community variance in the form of median odds ratios (MOR = 0.12), indicating a 12.0% lower likelihood of ever screened for cervical cancer of women from an average community (results not shown in Table 2).

**Table 2:** Individual and community-level determinants of cervical cancer screening

Fixed effects	Model 2 including individual-level determinants		Model 3 including community-level determinants	
	OR	(95% CI)	OR	(95% CI)
Individual-level characteristics				
Age group				
≤30 (Ref)				
31–49	2.01	(1.72–2.34)***	2.01	(1.72–2.34)***
Religion				
Christians (Ref)				
Non-Christians	0.93	(0.69–1.26)	0.94	(0.70–1.26)
Education level				
≤Primary school (Ref)				
Secondary school	1.36	(1.12–1.65)**	1.31	(1.08–1.59)**
Post-secondary	1.68	(1.27–2.23)***	1.63	(1.23–2.16)**
Employment status				
No (Ref)				
Yes	1.35	(1.17–1.55)***	1.35	(1.17–1.55)***
Covered by health insurance				
No (Ref)				
Yes	1.94	(1.61–2.33)***	1.95	(1.63–2.34)***
Ever used modern contraceptives				
No (Ref)				
Yes	1.54	(1.25–1.90)***	1.51	(1.22–1.86)***
Total children ever born				
≤3 (Ref)				
≥4	1.08	(0.91–1.29)	1.08	(0.90–1.29)
Age at first sex				
Never had sex (Ref)				
≤17	34.42	(14.92–79.36)***	34.66	(15.03–79.93)***
18–22	31.09	(13.52–71.51)***	30.99	(13.47–71.29)***
23–37	34.74	(14.86–81.24)***	33.81	(14.46–79.05)***
Wealth index				
Poorest (Ref)				
Poor	0.99	(0.71–1.38)	0.95	(0.68–1.32)
Middle	1.32	(0.96–1.81)	1.17	(0.85–1.62)
Rich	2.41	(1.78–3.24)***	1.52	(1.06–2.20)*
Richest	2.88	(2.09–3.96)***	1.64	(1.09–2.47)*
Amount of media exposure				
None (Ref)				
Single	1.11	(0.90–1.36)	1.12	(0.91–1.39)
Multiple	1.23	(1.01–1.54)*	1.27	(1.03–1.58)*
Household size				
1–4 (Ref)				
5–8	1.03	(0.90–1.18)	1.05	(0.91–1.20)
9+	1.00	(0.77–1.30)	1.03	(0.79–1.35)
Place of delivery				
Health facility (Ref)				
Home	0.49	(0.34–0.70)***	0.50	(0.35–0.72)***
Never given birth	1.00	(0.87–1.16)	1.00	(0.86–1.15)
Community-level determinants				
Women with favorable attitude towards IPV against women				
Low (Ref)				
High			1.21	(1.04–1.41)*
Women with high decision making autonomy				
Low (Ref)				
High			0.94	(0.80–1.10)
Women with a non-poor wealth index				
Low (Ref)				
High			1.54	(1.14–2.05)**
Difficulty in access to healthcare				
Low (Ref)				
High			0.81	(0.65–1.02)
Type of residence				
Rural (Ref)				
Urban			1.03	(0.76–1.40)

*p < 0.05; **p < 0.01; ***p < 0.001, Ref = Reference Category, OR = Odds Ratios, CI = Confidence Interval

After running the null model (model 1), level one fixed effects (individual-level covariates) were controlled for in model II. Results obtained in Model II depict statistically significant (p < 0.001) proportional change (−17) in community-level variance (39.0%). A reduction in the community-level variance implied a reduction in the proportion of the unexplained variance arising from differences in communities. A reduction in the community-level variance also indicated the community differences in the frequency of individual factors in Zimbabwe supporting the use of multi-level analysis as well. After running the model with random effects as well as level one fixed effects (model II), level two fixed effects (community-level predictors) were controlled for in the mixed effects model (model III). It was observed that the community-level variance reduced marginally in model III, suggesting similarity in the frequency of community-level determinants in all Zimbabwe communities. After controlling for individual-level and community-level factors, the variation in cervical cancer screening among communities remained significant.

Results from model II (comprised of only individual-level factors) indicate that the odds of cervical cancer screening were statistically significant, and higher among women age 31–49 years (OR = 2.01; 95% CI 1.72–2.34) than among their counterparts age $\le$ 30 years, with secondary (OR = 1.36; 95% CI 1.12–1.65) and post-secondary (OR = 1.68; 95% CI 1.27–2.23) compared with their counterparts with none or primary education, working (OR = 1.35; 95% CI 1.17–1.55) than non-working, covered by health insurance (OR = 1.94; 95% CI 1.61–2.33) than those without health insurance, ever used modern contraceptives (OR = 1.54; 95% CI 1.25–1.90) than those who never used modern contraceptives, and initiated sex [at $\le$ 17 years (OR = 34.66; 95% CI 15.03–79.93), 18–22 years (OR = 30.99; 95% CI 13.47–71.29), and 23–37 years (OR = 33.81; 95% CI 14.46–79.05)] than women who never had sex, rich (OR = 2.41; 95% CI 1.78–3.24) and richest (OR = 2.88; 95% CI 2.09–3.96) compared with their counterparts in the poorest wealth quintile, had exposure to multiple media (OR = 1.23; 95% CI 1.01–1.54) than those exposed to none. However, the odds of uptake of cervical cancer screening were lower among women who did not deliver from health facilities (OR = 0.49; 95% CI 0.34–0.70) than among those who delivered from health facilities. Additionally, the Variance Partition Coefficients or ρ (the Greek rho) (results not shown) revealed that 39.0% of the variance in cervical cancer screening was explained by common community characteristics (ρ = 0.39, p < 0.0001).

The results of model III (Table 2) indicate that the odds of cervical cancer screening were higher among women age 31–49 years (OR = 2.01; 95% CI 1.72–2.34) than among their counterparts age $\le$ 30 years, working (OR = 1.35; 95% CI 1.17–1.55) than non-working, with secondary education (OR = 1.31; 95% CI 1.08–1.59) and post-secondary education (OR = 1.63; 95% CI 1.23–2.16) compared with their counterparts with none or primary education, covered by health insurance (OR = 1.95; 95% CI 1.63–2.34) than those without health insurance, ever used modern contraceptives (OR = 1.51; 95% CI 1.22–1.86) than those who never used modern contraceptives, had exposure to multiple media (OR = 1.27; 95% CI 1.03–1.58) than those exposed to none, and rich (OR = 1.52; 95% CI 1.06–2.20) and richest (OR = 1.64; 95% CI 1.09–2.47) compared with their counterparts in the poorest wealth quintile. Considering age at first marriage, the odds of cervical cancer screening were higher among women whose age at first sex was $\le$ 17 years (OR = 34.66; 95% CI 15.03–79.93), 18–22 years (OR = 30.99; 95% CI 13.47–71.29), 23–37 years (OR = 33.81; 95% CI 14.46–79.05) than those who never had sex. However, the odds of uptake of cervical cancer screening were lower among women who did not deliver from health facilities (OR = 0.50; 95% CI 0.35–0.72) than among those who delivered from health facilities. Regarding community-level factors, the odds of cervical cancer screening were higher among women who were residing in communities with a high proportion of women with a favorable attitude towards IPV against women (OR = 1.21; 95% CI 1.04–1.41) than among those residing in communities with a low proportion with favorable attitude towards IPV against women and a non-poor wealth index (OR = 1.54; 95% CI 1.14–2.05) than those residing in communities with a low proportion of women with a non-poor wealth index.

## Discussion

To the best of our knowledge, this is the first study to describe the individual- and community-level determinants of the uptake of cervical cancer screening in Zimbabwe. We found a relatively low prevalence of cervical cancer screening, i.e., 13.4% among women of reproductive age in Zimbabwe. The individual-level factors that were positively associated with cervical cancer screening in Zimbabwe were middle aged women (31–49 years), secondary or higher school education, currently working, having health insurance, ever used modern contraceptives, given birth to ≥ 4 children, ≥ 17 years age at first sex, women from rich or highest wealth/income quintile, and exposure to multiple media sources. The community-level factors positively associated with the uptake of cervical cancer screening were living in communities with high proportion of women with decision making autonomy; favorable attitude towards IPV against women, and non-poor wealth index.

Previous studies have adopted a more narrow geographical scope as they focused on single/specific locations and considered only individual level variables [17–19, 22]. Our study provides unique data by analyzing both individual and community level factors and its association with the uptake of cervical cancer screening using multi-level regression analyses. The prevalence of cervical cancer screening found in our study was low. However, previous studies from Shamva district, Mashonaland central province, Zimbabwe [20], and Hurungwe rural district, Mashonaland West province, Zimbabwe [16] reported much lower rates of cervical cancer screening: 9% and 5%, respectively. The much lower rates reported by earlier studies point to an upward trend and improvement at the population level in cervical cancer screening over the last decade. The difference in results in our study and previous studies could be explained by the differences in the sampling frame, and time-point of analysis. Prior studies from South Africa [42] and Botswana [43] reported relatively higher proportions of women who had ever screened for cervical cancer: 52% and 72%, respectively.

Our study revealed that both individual- and community-level factors influence the uptake of cervical cancer screening. Higher odds of cervical cancer screening were associated with socio-demographic factors of the reproductive age women in Zimbabwe. First, middle-aged women (31–49 years) were significantly associated with higher odds of ever screened for cervical cancer. This study finding is consistent with earlier studies from Harare, Zimbabwe [22], and Kenya, which found the odds of ever screened for cervical cancer were higher among middle-aged women (aged 35–49 years) than among younger females (aged 15–24 years) [34]. Furthermore, older women were significantly associated with higher odds of ever screened for cervical cancer than were younger women in Portland, Jamaica [44], Ugrachandi Nala, Kavre, Nepal [45], and Eastern China [46]. These data suggest that middle-aged to older women, after experiencing early signs and symptoms of cervical cancer or exposure to other illnesses, may be motivated to seek screening for cervical cancer [47, 48]. Therefore, it is important to advocate for targeted screening and inform younger women about the benefits of cervical cancer screening, particularly in rural and disadvantaged populations.

Our data highlights that a higher proportion of working women reported ever screened for cervical cancer compared with non-working women, which is consistent with previous reports [27, 34]. The employment of women contributes to their financial independence and to an improvement in the reproductive health care services [36]. Likewise, women covered by health insurance had a higher likelihood of ever screened for cervical cancer, which corroborates with prior reports from Africa and elsewhere [34]. The cost of screening and ensuing treatment is perceived as a major barrier to the uptake of cancer screening programs [15]. Given that the majority of the women (89%) in Zimbabwe are not covered by health insurance [27], we recommend increased investment in strengthening both the primary care system and the provision of universal health insurance coverage to have equitable and affordable care for all women.

Furthermore, this study highlights that women who delivered at health facilities had a higher probability of ever screened for cervical cancer, which is consistent with studies from other regions in Sub-Saharan Africa [34]. It is likely that women who had contact with the health care providers may have perceived cervical cancer screening positively. Institutional deliveries should therefore be encouraged in Zimbabwe, as the perceived benefits could extend beyond pregnancy related outcomes [31, 49, 50]. Our data also indicated higher odds of cervical cancer screening among married women versus those never married. Typically, in Zimbabwe, the age at first marriage coincides with age at first sex and therefore also with potential first exposure to HPV. Almost all HPV infections occur within 3–4 years prior to first sexual intercourse [51, 52]. Therefore, as per recommendations for cervical cancer screening by the American Cancer Society [53], we recommend the local government to initiate cervical cancer screening at younger ages or at least within three years of marriage or sexual intercourse. In this study, the exposure to multiple media sources was found to be significantly associated with an increased uptake of cervical cancer screening, as also found elsewhere in Kenya [34]. The digital and print media are common channels through which health information is accessed [54], and therefore, various media platforms can be leveraged to empower women and improve their access to health information to facilitate informed health choices.

In this study several community-level factors were also significantly associated with the uptake of cervical cancer screening. First, the odds of cervical cancer screening were higher among women who were residing in communities with a high distribution of women with a non-poor wealth index than among those residing in communities with a low distribution of women with a non-poor wealth index, which is consistent with previous study from Zimbabwe [20] and Eastern Jamaica [44]. However, another study from Kenya found no significant relationship between cervical cancer screening and the overall wealth index of communities [34]. This finding points to the importance of addressing factors responsible for economic disparities at the community-level. Surprisingly, the cervical cancer screening was higher among women residing in communities with favorable attitude towards IPV against themselves. The probable explanation for this finding is that women’s pre-supposition of the natural superiority of men over women results in a rejection of their own freedom and rights; women’s acceptance of subordinating cultural views is an attribute of patriarchal settings [55, 56]. Slavery to patriarchal sentiments is a manifestation of marital dependence/disempowerment [57]. However, there is lack of evidence regarding marital status and cervical cancer screening, calling for more research in this area. A study in Kenya did not establish a significant relationship between the attitude of women towards IPV against women and cervical cancer screening [34].

The overall low uptake of cervical cancer screening in Zimbabwe compared with other countries could be attributed to supply- as well as demand-side barriers to health care services. On the supply side, the crumbling economy of Zimbabwe has affected the health sector; for instance, it resulted in the suspension of pap smears from the routine care menu for women in the late 1990s due to the inability to sustain the manpower and infrastructural requirements for the program [58]. The cervical cancer screening provision was dependent on private providers, which is cost prohibitive; the cost of cervical cancer screening in private clinics is seven times the cost for the same service at public health facilities [15]. Some of the demand-side barriers of cervical cancer screening reported by previous studies include limited knowledge (20%) of cervical cancer screening [20]. Our study findings point to the need for a self-pap-smear-collection strategy. This is because, the self-sampling strategy can provide sensitivity comparable with clinician-collected-samples. Additionally, the self-sampling strategy is well tolerated by women in the country [59]. Our study findings also suggest addressing both supply and demand side barriers; addressing supply related barriers to cervical cancer screening would call for measures to resuscitate the economy in order to create direct as well as spillover effects for the health sector in general and for the cervical cancer screening program in particular [60]. Increased investment in healthcare and particularly for cervical cancer screening programs should be accompanied with information about cervical cancer screening benefits for younger women which may further improve the uptake of cervical cancer screening [24, 61].

## Limitations of the study

This study has several limitations. First, given the nature of study design and the use of cross-sectional data, our analysis does not allow us to determine causation, but only provide evidence of association between individual- and community-level factors and the uptake of cervical cancer screening. Second, due to lack of relevant data, we could not assess the role that supply-side factors, such as the availability and quality of health care services, which are known to influence the uptake of cervical cancer screening. Third, the sampling error may have affected the precision of the findings, since the study did not use census data. However, enumeration areas/clusters were selected from the whole country to ensure both representativeness of data and precise findings. Community-level variables were derived by aggregating individual responses to their respective clusters with an assumption of cluster homogeneity. Therefore, associations at aggregated levels do not directly apply to individuals but to a group of individuals in each cluster, which calls for an interpretation of the findings with such considerations. Fourth, the lack of data on the social and environment context within the community limited our understanding of the total variance in cervical cancer screening; cervical cancer screening explained by the current study excludes the effects of such important community-level factors, for instance, a relative lack of opportunities (such as jobs) and weak social networks. Nonetheless, the multi-level regression analyses successfully isolated individual-level from community-level effects with reference to cervical cancer screening in Zimbabwe. The intra-cluster correlation coefficient (ICC) provided estimates for the variation in the components of the multi-level regression analyses, notwithstanding the fact that it does not precisely indicate the extent of similarity in terms of ratings of women dwelling in the same village; the (within-group) interrater agreement. However, the coefficient sheds light regarding the dependability of scores of participants in their respective clusters [38].

## Conclusions

Our study demonstrated that both individual- and community-level factors influenced the uptake of cervical cancer screening in Zimbabwe. The study results underscored the need for policy measures for rolling out community-level economic empowerment programs targeted at women to enhance the uptake of cervical cancer screening and to improve their overall health. The universal national health insurance policy remains an aspirational goal to enhance women’s access to healthcare services, including cervical cancer screening.

## Data Availability

Data are from the Demographic and Health Survey. The dataset is open to qualified researchers free of charge. To request access to the dataset, please apply at http://dhsprogram.com/data/Access-Instructions.cfm.

## Abbreviations

CI: Confidence interval
FDA: Food and Drug Administration
HPV: Human papillomavirus
OR: Odds ratio
SDGs: Sustainable Development Goals
SES: Socio-economic status
